# A real-time, bioluminescent annexin V assay for the assessment of apoptosis

**DOI:** 10.1007/s10495-018-1502-7

**Published:** 2018-11-29

**Authors:** Kevin Kupcho, John Shultz, Robin Hurst, Jim Hartnett, Wenhui Zhou, Thomas Machleidt, Jamison Grailer, Tracy Worzella, Terry Riss, Dan Lazar, James J. Cali, Andrew Niles

**Affiliations:** 1 Promega Corporation, 2800 Woods Hollow Road, Madison, WI 53711 USA; 2 Promega Biosciences LLC, 277 Granada Drive, San Luis Obispo, CA 93401 USA

**Keywords:** Annexin V, Phosphatidylserine translocation, Real-time apoptosis, Luciferase complementation, Necrosis, Bioluminescent imaging

## Abstract

Apoptosis is an important and necessary cell death program which promotes homeostasis and organismal survival. When dysregulated, however, it can lead to a myriad of pathologies from neurodegenerative diseases to cancer. Apoptosis is therefore the subject of intense study aimed at dissecting its pathways and molecular mechanisms. Although many assay methods exist for confirming whether an apoptotic response has occurred in vitro, most methods are destructive and involve laborious operator effort or specialized instrumentation. Here we describe a real-time, no-wash, microplate method which utilizes recombinant annexin V fusion proteins containing evolved binary subunits of NanoBiT™ luciferase. The fusion proteins, a time-released enzymatic substrate, a necrosis detection dye and exogenous calcium ions are delivered via an optimized and physiologically inert reagent directly to cells in culture at the time of treatment or dosing. Luminescent signals proportional to phosphatidylserine (PS) exposure and fluorescent signals generated as a result of loss of membrane integrity are then collected using a standard multimode plate reader at scheduled intervals over the exposure. The resulting luminescent and fluorescent data are then used to define the kinetics and magnitude of an apoptotic response. This study details our efforts to develop, characterize, and demonstrate the features of the assay by providing relevant examples from diverse cell models for programmed cell death.

## Introduction

Multicellular organisms have evolved a programmed cell death also known as apoptosis [1] which operates throughout development and as part of the immune system to eliminate damaged, infected, aged or redundant cells [2]. Perturbations to this process are linked to a multitude of developmental defects and disease [3]. Therefore, modulation of apoptosis provides a target rich environment for therapeutic intervention.

Currently, a majority of research efforts for apoptosis are conducted in vitro using cell based models. This work is possible because the principle molecular mechanisms responsible for execution of the program can be recapitulated with primary or immortalized cell lines [4]. Furthermore, these models lend themselves to near limitless experimental manipulation.

Although more efficient and expeditious than in vivo models, scientific challenges also exist for plate-based culture methods. For instance, in vitro cytotoxicity is a multi-faceted phenomenon intrinsically linked to the potency of the stimulus and duration of the exposure. The precise mechanism by which cells lose functional viability is further dependent upon the mechanism of action of the stimulus, as well as intrinsic features of cellular susceptibility relating to lineage, cell cycle and metabolic or genetic status. Depending upon these variables, cell death may occur via oncotic necrosis, apoptosis, or other forms of regulated cell death [5, 6]. Therefore, it is often experimentally difficult to establish a definitive mode of action for any compound or treatment without conducting orthogonal/complementary cell health assays on multiple cell types treated with broad dose coverage in thoroughly defined time courses [7, 8]. Furthermore, definitive markers of apoptosis, such as caspase activation, suffer from inherent enzymatic instability that, depending upon when assays are employed, can complicate the interpretation of data and lead to erroneous conclusions [9].

Live cell assay methods are highly desired because they potentially enable the detailed kinetic analysis of different cytotoxic pathways. Label-free methods utilizing electrical impedance (real-time cell analysis, RTCA), for instance, are able to monitor cytostasis and cytotoxicity in real time yet lack the ability to distinguish the different modes of cell death [10]. Substantial improvements in traditional microscopy methods, utilizing traditional and/or novel probes (e.g., pSIVA) paired with integrated optical collection, allow for retrospective analysis of image data by algorithms based on morphology and fluorescence parameters [11, 12]. Unfortunately, these platforms are limited by their requirement for specialized instrumentation, training and support, and their lack of throughput.

We endeavored to develop a simple, plate-based, homogenous, live cell apoptosis detection assay which is HTS-compatible and enables real time analysis. For this we chose PS exposure as our candidate marker because it is a well-validated marker for the early stages of programmed cell death [13].

Phosphatidylserine (PS) is an integral component of the plasma membrane that is actively confined to the inner membrane leaflet in healthy cells [14–16]. PS translocates to the outer leaflet of the plasma membrane during apoptosis where it is typically measured by fluorescently-labelled annexin V conjugates [17]. Annexin V is a preferred probe for PS exposure because of its high, calcium-dependent affinity and selectivity for the lipid [18]. Unfortunately, excess unbound fluorescent annexin V probe must be removed by repeated washes so that it does not confound determination of actual PS exposure by adversely contributing to background.

Our approach entailed the use of annexin V fusion proteins containing individual subunits of the NanoLuc binary technology (NanoBiT™). This newly described structural complementation reporter seemed particularly suitable due to sensitivity and large dynamic range [19]. Because the NanoBiT subunits were designed to exhibit low intrinsic affinity, their complementation would be largely driven by the PS dependent interaction of annexin V. Therefore, Annexin V-NanoBiT™ fusion proteins theoretically could be added directly to cells in culture without the need for any wash steps to control PS independent background signal. Another critical component was the development of a protected time-released luciferase substrate which enabled real-time readings over physiologically relevant time periods. Lastly, the incorporation of a cell-impermeant pro-fluorescent probe with high affinity for exposed DNA allowed for differentiation between apoptosis and necrosis [20].

## Materials and methods

### Reagents

Digitonin, paclitaxel, calcium chloride, 0.4% trypan blue, dimethyl sulfoxide and 7-AAD were purchased from Sigma-Aldrich. Panobinostat and bortezomib were sourced from Selleckchem. rhTRAIL was purchased from Gibco and staurosporine obtained from LC Laboratories. The Alexa Fluor® 488 Annexin V/Dead Cell Apoptosis Kit was purchased from Thermo-Fisher. Caspase-Glo®-3/7 Assay, digitonin solution and NanoGlo® Luciferase Assay Substrate were obtained from Promega. The pro-furimazine NanoBiT™ substrate (endurazine) and a novel asymmetric cyanine dye, Necrosis Detection Reagent, were synthesized and purified by Promega Biosciences, LLC.

### Annexin V-fusion proteins

Full-length annexin V-fusion proteins possessing complementary and molecularly evolved subunits of the binary luciferase reporter NanoBiT™ were cloned and expressed using high-density induction. The Annexin V-LgBiT and Annexin V-SmBiT recombinant proteins were each purified to greater than 95% purity using a combination of standard and proprietary chomatographic techniques. The highly concentrated protein pools were quantified by optical density (and confirmed by Coomassie-stained SDS-PAGE gels), 0.22 µm filtered, then supplemented with autoclaved glycerol to 50% v/v. Aliquots were stored at − 20 °C.

### Cell culture

Raji, K562, DLD-1, SK-ES-1, HepG2, A549, PC-3 and HeLa cells were obtained from American Type Culture Collection (ATCC) with supporting documentation of authenticity. Unless noted otherwise, all cells were used at a density of 10,000 cells/well in white 96 well plates (Costar). Ham’s F-12, RPMI-1640, McCoy’s 5A, DMEM, CO_2_-independent medium and 0.05% trypsin were obtained from Gibco. Fetal bovine serum was purchased from Seradigm. All cells were routinely passaged to maintain robust cell health (≥ 95% viability by trypan blue exclusion) in a humidified atmosphere at 5% CO_2_ at 37 °C.

### Bioluminescent annexin assay reagent optimization

DLD-1 cells were seeded into two assay plates and allowed to attach. One plate received 300 ng/ml rhTRAIL, whereas the other received a volume-matched, vehicle control. Both plates were returned to an incubator for a 3.5 h exposure. The complementary Annexin-NanoBiT™ fusion proteins were subjected to a titration matrix in complete McCoy’s medium supplemented with furimazine and 1 mM CaCl_2_. The twofold, titrated pairs were delivered to both plates as a 2× reagent, and luminescence associated with productive PS-binding and resulting NanoBiT™ pairing was collected using a BMG POLARstar®. The induced/uninduced luminescence ratio was used to guide the determination of optimal pairing concentrations.

### Sustained-release NanoBiT™ substrate

Raji, K562, DLD-1 and SK-ES-1 cells were harvested, washed and adjusted to 100,000 viable cells/ml in CO_2_-independent medium + 10% FBS. Digitonin was applied to the cells at a final concentration of 30 µg/ml to facilitate immediate and complete PS exposure by a primary necrosis mechanism. Replicates of each cell type were added to a 96 well plate in 100 µl volumes. An optimized formulation of Annexin–NanoBiT™ fusion proteins was prepared in CO_2_-independent medium + 10% FBS supplemented with 1 mM CaCl_2_ and NanoBiT™ substrate (endurazine) and applied as a 2× reagent. Luminescence was collected every hour for 16 h at room temperature using a BMG POLARstar®.

Next, K562 and Raji were twofold serially diluted in RPMI 1640 + 10% FBS, respectively, and plated at densities ranging from 312 to 20,000 cells per well. Both plates were dosed with 500 nM bortezomib or vehicle control in the presence of the complete real-time annexin V reagent containing the NanoBiT™ substrate. Luminescence and fluorescence were collected in real-time during a 48 h exposure using a BMG CLARIOstar®. Data was plotted as net luminescence (induced–uninduced) for each cell concentration.

### Real-time PS exposure (and loss of membrane integrity) kinetics

Dose-escalating dilutions of three mechanistically-distinct cytotoxins were applied to three different cell types: staurosporine was added to PC3, paclitaxel contacted with A549, and DLD-1 subjected to panobinostat. In all instances, the 2× bioluminescent annexin reagent was immediately added after reagent preparation in respective growth media with 10% FBS, supplemented with 1 mM CaCl_2_ and necrosis detection reagent, to initiate the assay time course. Luminescence and fluorescence were sequentially collected throughout the compound exposures either by incubation in a humidified incubator followed by manual transport to the multimode reader or by using a BMG CLARIOstar® equipped with atmospheric control programmed to collect data automatically.

### Annexin V reagent tolerability

HepG2 and K562 cells were dosed with serial dilutions of paclitaxel and bortezomib, respectively, for 48 h with either the bioluminescent annexin assay reagent with necrosis detection reagent or with the necrosis detection reagent alone to gauge potential toxicity contributed by the annexin assay reagent. Fluorescence associated with changes in membrane integrity was collected at time points relevant for the onset of cytotoxicity for each treatment.

### High throughput compatibility

A Combi nL (ThermoFisher) liquid dispenser was used to deliver K562 cells (2000 cells/well) to 336 wells of a white, 384-well plate (Greiner). A cell-free, culture medium control was added to 48 wells to serve as reagent background control. Either 2 µM bortezomib or vehicle control was added to replicate wells. Finally, the bioluminescent annexin reagent and necrosis detection reagent were added to all wells using the automated dispenser. Fluorescence and luminescence data were collected in real-time using a Tecan Spark® 20M equipped with atmospheric control for 24 h and at a final 48 h endpoint. Z′ data were calculated as per Zhang et al. [21].

### Flow cytometry

K562 cells were dosed with either 0.01, 0.1, or 10 µM bortezomib or a vehicle-matched excipient in RPMI-1640 + 10% FBS for 16 h and 25 h. Following the exposure, 1 million cells were harvested, washed and labelled with Alexa Fluor® 488-Annexin V and 7-AAD as directed by the manufacturer. The samples were analyzed by a guava easyCyte® 8HT (Millipore) with 10,000 events collected for each sample. In parallel, 10,000 K562 cells per well were dosed with a threefold dilution series of bortezomib in the presence of the bioluminescent annexin and necrosis detection reagent. Luminescence and fluorescence were collected in real-time every hour using a BMG CLARIOstar® equipped with an ACU for 25 h.

### Caspase activation

HepG2 cells were exposed to serial dilutions of paclitaxel in DMEM + 10% FBS in the presence of the bioluminescent annexin and necrosis detection reagent. Luminescence and fluorescence data were gathered at 1, 2, 3.5, 6.5, 24 and 30 h exposure time points by removing the plate from a 37 °C, 5% CO_2_ incubator and employing a GloMax® Discover multimodal plate reader (Promega). The plate was returned to the incubator for additional exposure after each time point. Six parallel plates were exposed to paclitaxel as above but processed at the designated time points by the addition of Capase-Glo®-3/7 Assay reagent. Luminescence was collected at each time point using a GloMax® Discover.

### Bioluminescent imaging

HeLa cells were seeded into sterile, 35 mm imaging dishes (ibidi) in DMEM + 10% FBS and allowed to attach overnight. The medium was carefully removed and replaced with CO_2_-independent medium (Gibco) containing 10% FBS and the complete bioluminescent annexin reagent. After 2-h of pre-incubation at 37 °C, either staurosporine (2 µM final concentration) or a vehicle matched control volume were added. Brightfield and bioluminescence images were collected every 60 s for a period of 6 h using a LV-200 Bioluminescence Imaging System (Olympus) equipped with a 100x/1.4 NA objective, a Hamamatsu imagEM EMCCD camera and a heated stage. The luminescence signal was gathered at an electro-multiplying gain of 600 using a 3 s exposure time. Post-acquisition image processing was performed using FIJI and included linear contrast adjustment and conversion into mp4 file format.

## Results

### Optimized parameters for the bioluminescent annexin reagent

**Fig. 1:**
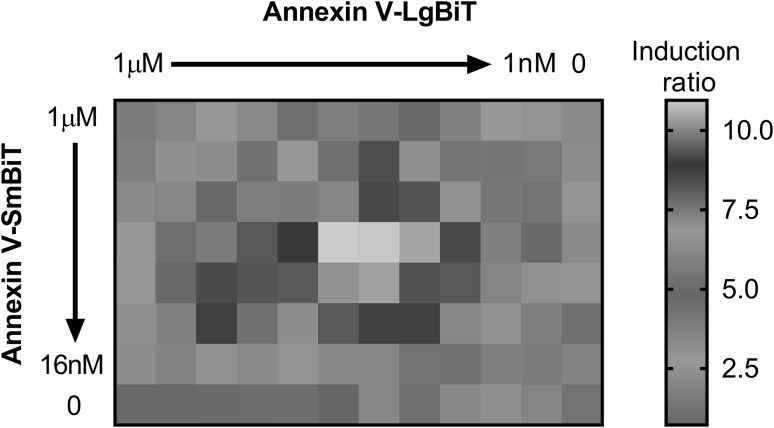
Annexin V-LgBiT and Annexin V-SmBiT fusion proteins displayed stoichiometric- and concentration-dependence in the assay. A twofold matrix titration of the proteins revealed a near equimolar optima of 30 nM and 60 nM for Annexin V-LgBiT and Annexin V-SmBiT, respectively, for PS binding and NanoBiT™ complementation

**Fig. 2:**
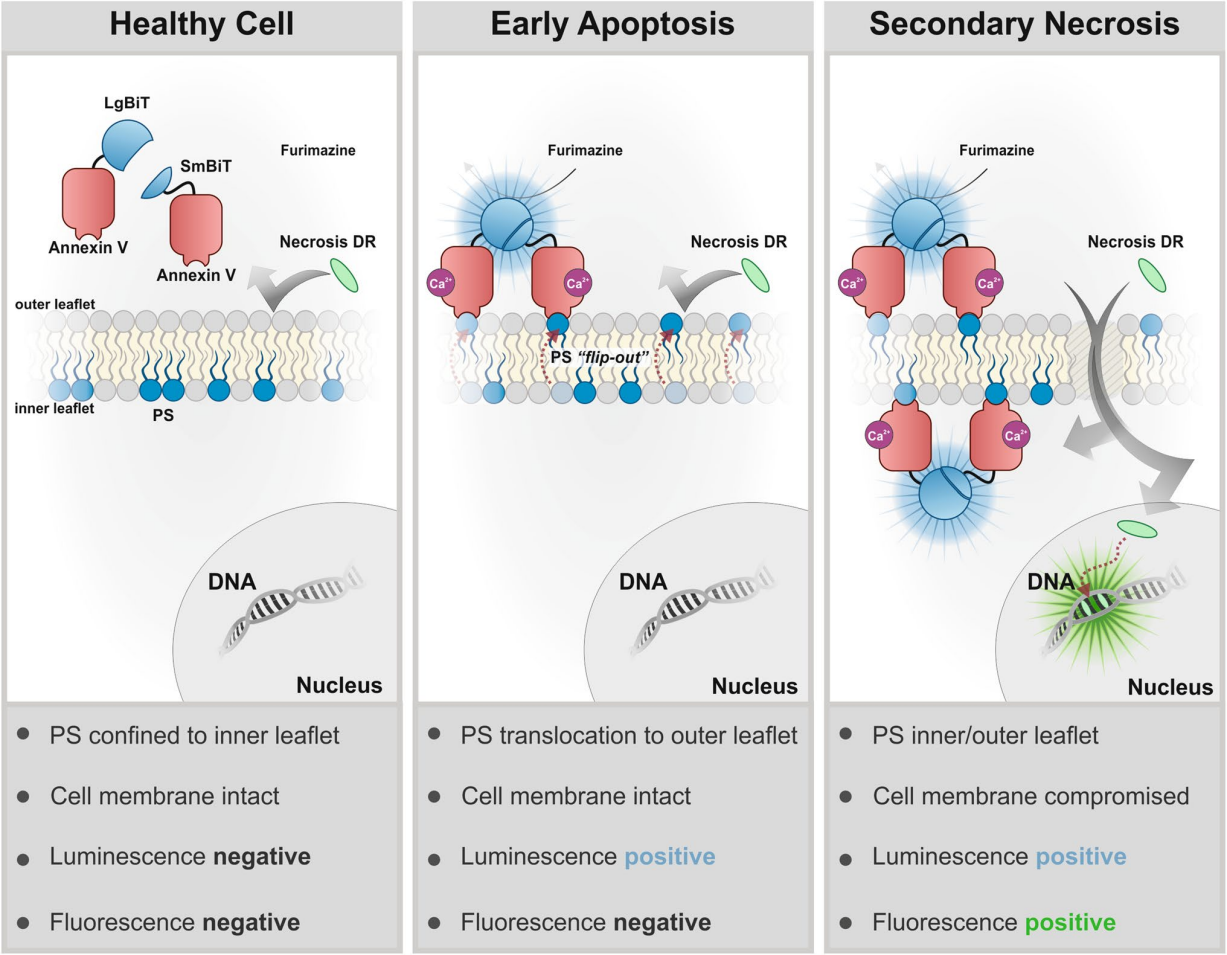
The bioluminescent annexin assay concept. Healthy cells produce no necrosis fluorescence and few productive annexin binding events. During early apoptosis, PS exposure leads to Annexin V-LgBiT and Annexin V-SmBiT binding at the membrane surface. The reconstituted NanoBiT™ enzyme produces luminescence in the presence of the furimazine substrate product. A viable cell membrane precludes Necrosis Detection Reagent from staining cellular DNA. Productive Annexin V-NanoBiT™ pairing occurs on the outside and inside of damaged cell membranes during secondary necrosis as does productive staining of the nucleus by the Necrosis Detection Reagent.

A matrix titration experiment designed to examine Annexin V-LgBiT and Annexin V-SmBiT pairing dynamics revealed a stoichiometric dependence in the sub-micromolar range (Fig. 1). When the test components were delivered to rhTRAIL or vehicle treated DLD-1 cells, near equimolar concentrations of the fusion proteins led to optimal TRAIL-induced binding and subsequent productive NanoBiT™ pairing. These near-equimolar ratios yielded the brightest net luminescence. Fourfold shifts in fusion protein concentration ratios away from the near-equimolar concentrations led to nonproductive pairing after PS binding and diminished luminescence. Likewise, the absolute final concentrations of the fusion proteins contacted with apoptotic and non-apoptotic cells were important. Concentration increases substantially above optimal increased non-specific background (random collisional pairing in solution) of the proteins and diminished the induced to un-induced assay window. Decreases in final concentrations below optimal reduced luminescence output while also reducing the background. Based on these findings, the purified proteins were thereafter adjusted and separately stored as reagent components at 1000× of their respective optimal final assay concentrations. This titrational series reinforced our conceptual understanding of the homogeneous annexin V complementation assay concept and provided a more formal framework describing the interactional interface (Fig. 2).

### Time-released NanoBiT™ substrate

Standard endpoint applications utilizing NanoLuc™ or NanoBiT™ luciferases employ a chemical derivative of coelenterazine (furimazine) as the optimal substrate. Furimazine has limited stability in aqueous solutions which limits its suitability for real-time measurements. A methyl pivalate ester-protected furimazine (endurazine) circumvents this limitation by slowly releasing furimazine upon esterase hydrolysis from live cells. In order to gauge the utility of this novel NanoBiT™ substrate for use in real time assays, we needed to examine: (1) the esterase-dependent rate of product formation for different cell types, (2) the inherent esterase stability and continued conversion of the pro-substrate after cell death and (3) the luminescent signal magnitude and proportionality resulting from these de-protection events.

**Fig. 3:**
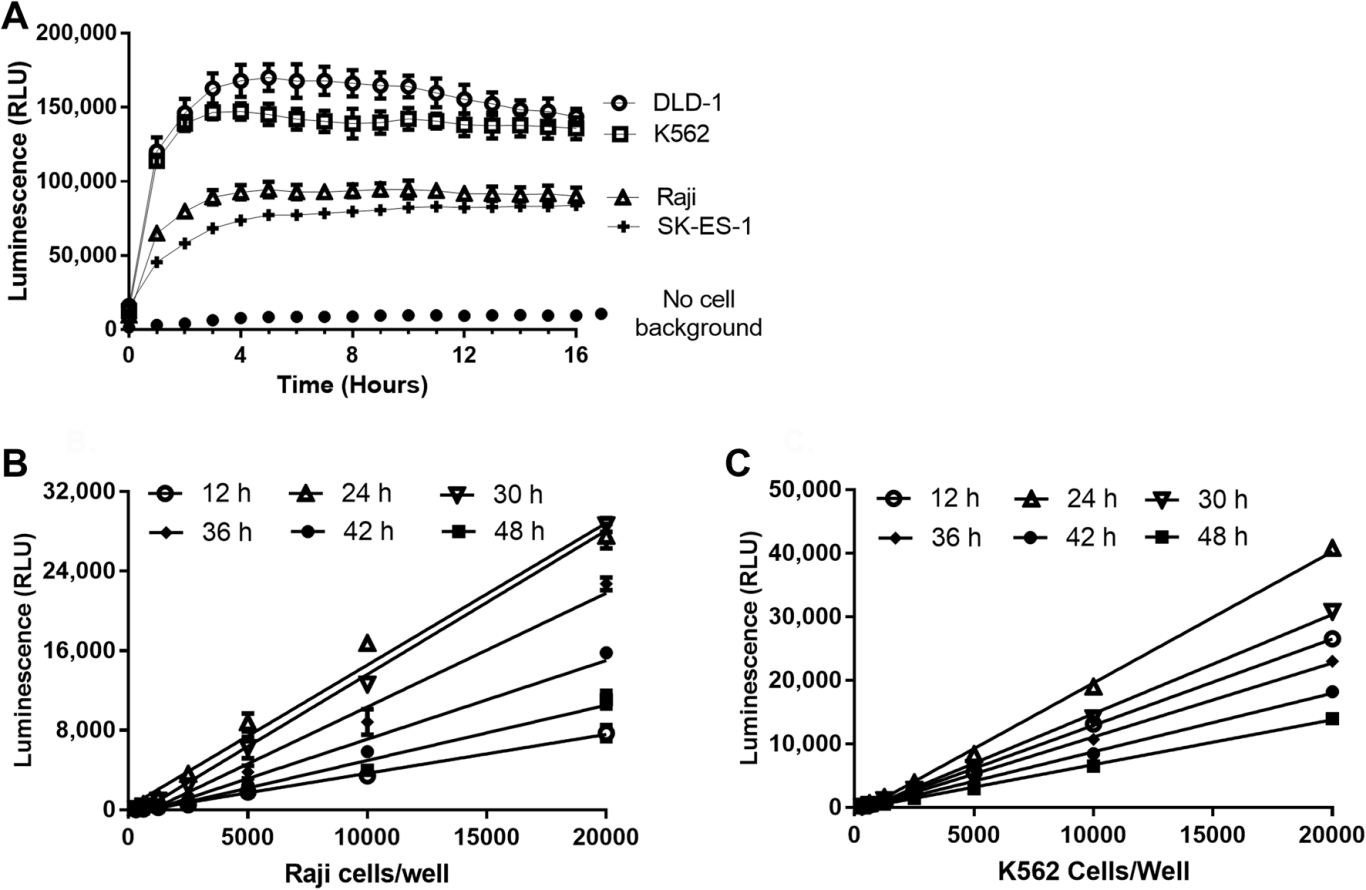
Characterization of time-released luciferase substrate. Esterase-dependent cleavage rate of the pro-furimazine substrate is cell line dependent (**a**). The cell lines were adjusted to 10,000 cells per well and permeabilized via digitonin treatment, releasing endogenous esterases and exposing PS. The annexin assay components were delivered to assay wells and luminescence collected as a function of time. Endurazine deprotection by esterase activity was non-limiting for Raji and K562 and allowed saturation of the PS-complemented Annexin V-NanoBiT® fusion pairs throughout early and late execution of the apoptotic program (**b**). Decay in total luminescence was observed as cells progressed to secondary necrosis (at 36 and 30 h, respectively), but proportionality of PS exposure was conserved throughout 48 h

Inherent esterase activity varied within Raji, K562, DLD-1 and SK-ES-1 cells by as much as twofold, with DLD-1 and K562 cells having the highest cleavage potential (Fig. 3a). Although Raji and SK-ES-1 cells contained less esterase (or less of the preferred isoforms for optimal pro-substrate conversion), the magnitude of signals generated were sufficient enough to support further assay development activities by being in excess of eight to tenfold over background. More importantly, endurazine provided a steady, persistent, “glow-type” luminescence to at least 16 h post cell death. Interestingly, a steady state between product formation and product consumption was not achieved until about 4 h after contact of cells with pro-substrate at 37 °C. K562 and Raji, representative of “higher” and “lower” pro-substrate turnover/release potential cell lines identified above, were further examined to determine if esterase-dependence limited assay signal duration or proportionality to PS exposure. In these respects, both cell lines produced luminescent signals proportional to PS exposure by apoptosis throughout the 48 h time course (Fig. 3b). During the progression through secondary necrosis however, luminescent proportionality was maintained, but overall signal declined as esterase cleavage potential decayed.

### PS exposure and secondary necrosis as a function of compound dose and time

**Fig. 4:**
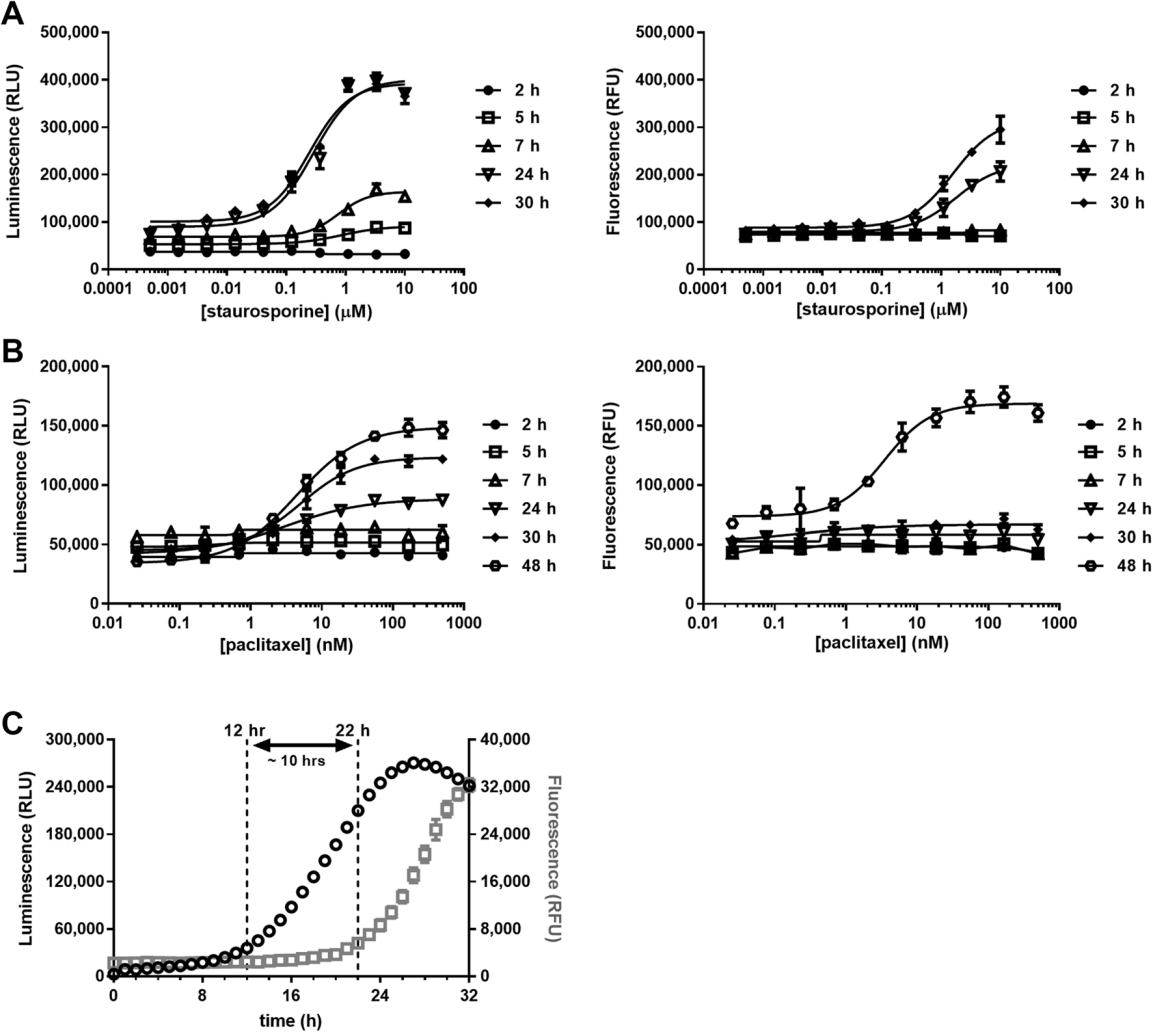
Kinetic profiles of PS exposure (luminescence) and loss of membrane integrity (fluorescence). Dose- and time-dependent maturation of apoptotic responses of **a** PC3 cells to staurosporine and **b** A549 cells to paclitaxel. The apoptotic phenotype produces a clear temporal lag between PS exposure and secondary necrosis (**c**) with panobinostat treated (3 µM) DLD-1. The data shown in this figure represent the mean ± SD of four replicate samples for each dose

The pleiotropic kinase inhibitor staurosporine initiated a luminescence response from PS exposure in PC3 cells beginning at 5 h with doses in the micromolar range without commensurate increases in dead cell fluorescence (Fig. 4a). The magnitude of the luminescent signal grew steadily at 7 h owing to more PS exposure in the wells by progression of the apoptotic program, again without appreciable secondary necrosis. By 24 h, the full potency and efficacy (magnitude of response) of staurosporine was achieved with respect to PS exposure, but now with a measurable cell death fluorescence. Finally, cell death as a consequence of late-stage apoptosis was maximized at 30 h owing to completion of the cell death program.

A549 cells produced a different kinetic signature when exposed to the mitotic poison, paclitaxel (Fig. 4b). In this example, PS exposure was provoked after about 24 h of exposure and became fully mature between 30 and 48 h. The necrosis detection reagent indicated negligible cell death up through 30 h treatment, but significant cell death at 48 h, consistent with emergence of secondary necrosis.

The histone deacetylase inhibitor, panobinostat, produced dose- and time-dependent increases in PS exposure and necrosis fluorescence with DLD-1 cells during a 48 h exposure (data not shown). The kinetic relationship between PS exposure and loss of membrane integrity is most pronounced, however, when graphed as a single compound concentration versus time (Fig. 4c). At 3 µM, panobinostat initiated PS exposure at 12 h which was maximized after 26 h of exposure. Dead cell fluorescence due to secondary necrosis began at about 22 h. This 10 h kinetic lag between initial PS exposure and subsequent loss of membrane integrity is consistent with the progression of apoptosis and can be used as a line of evidence for defining mechanism of cell death.

### Real-time reagent tolerability

Because any real-time, cell health assay reagent requires co-incubation with vehicle and test compound treated cells over the entire study exposure period, it is of paramount importance to identify if the reagent itself confers any cytotoxic liability. We chose to study this by comparing the fluorescence profiles and progress curves obtained from our cell impermeant, non-cytotoxic DNA-binding dye (Necrosis Detection Reagent) in the presence or absence of the real-time annexin assay reagent in two model systems utilizing either an attachment-dependent or suspension cell line.

**Fig. 5:**
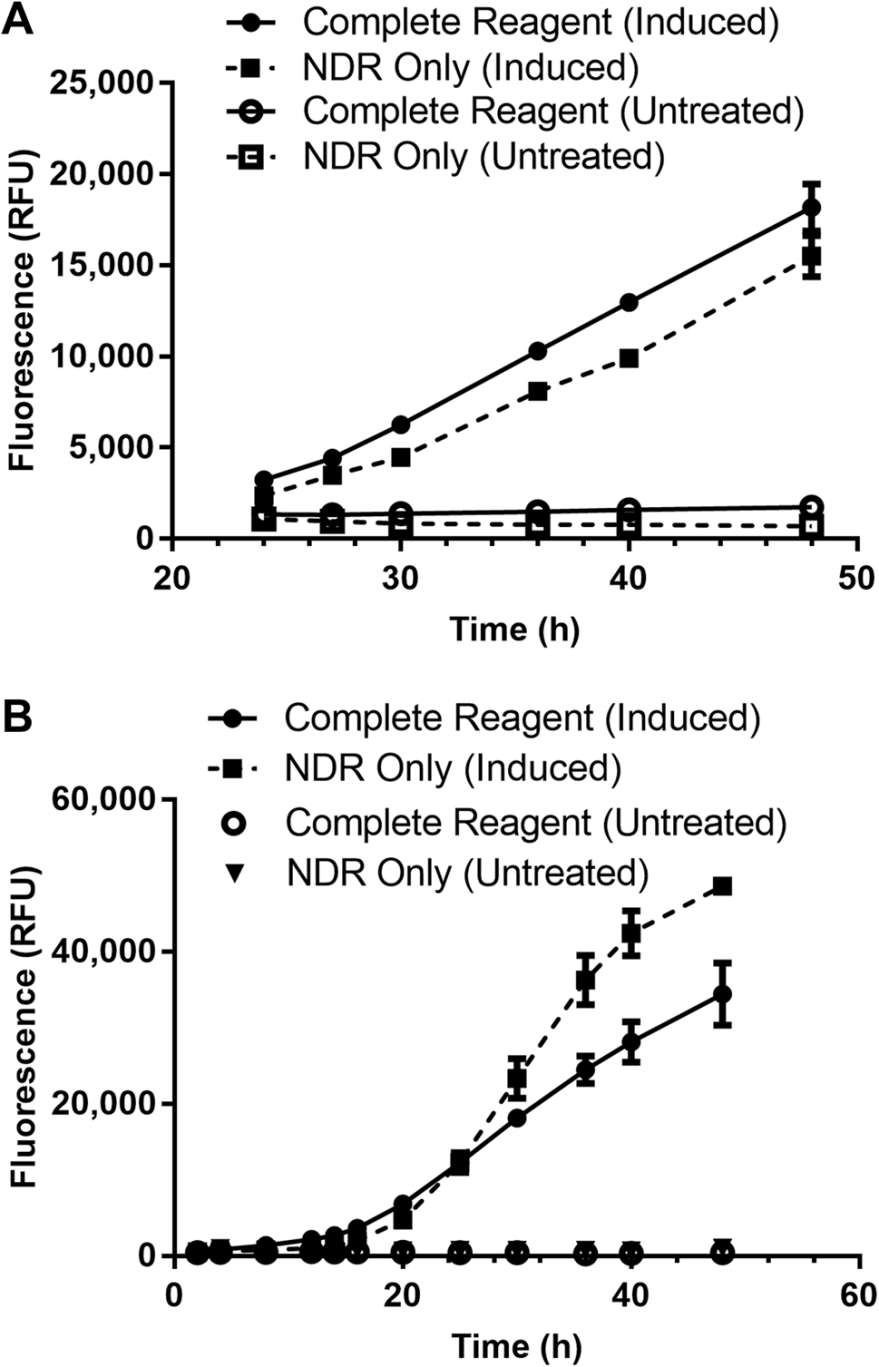
General tolerability of the real-time annexin reagent with and without treatment in extended exposures. HepG2 contacted with paclitaxel (**a**) or K562 exposed to bortezomib (**b**) in the presence of the complete annexin reagent with a biologically inert necrosis detection reagent (NDR) or the dye alone. The data shown in this figure represent the mean ± SD of four replicate samples for each time point

In the first model, HepG2 cells were exposed to a dose-range of paclitaxel for 48 h, with fluorescence collected in 3–8 h increments during the exposure. No appreciable differences existed in the kinetics of paclitaxel cytotoxicity, but a minor enhancement in fluorescence was observed in treated and untreated vehicle controls in the presence of the annexin assay reagent (Fig. 5a). Paradoxically, the annexin assay reagent produced a modest reduction in fluorescence relative to control when co-incubated with K562 cells dosed with bortezomib for 48 h (Fig. 5b). These types of subtle, but often contradictory influences have been reported previously for annexin reagent systems [22, 23].

### Assay performance in high-density formats

The practical utility of any cell-based assay method for use in drug discovery is dependent upon its scalability into high-density formats. Therefore, we examined high-throughput “fitness” using K562 cells, bortezomib and the complete annexin assay reagent delivered to 384-well microplates using automated dispensing. The luminescent annexin measure delivered a Z′≥0.5 upon apoptotic induction at 13 h and sustained this window to at least 24 h with 2 µM bortezomib (Table 1). These Z′ values represented from 4.4-fold (at 13 h) to 11.4-fold (at 24 h) increases in the apoptosis induction window. Due to the kinetics of secondary necrosis and a temporal lag in cell death after PS exposure, the measure of loss of membrane integrity by the fluorescence probe reached a Z′ ≥ 0.5 after 23 h and was maintained throughout the remainder of the 48 h exposure. Therefore, the temporal discordance in the luminescent and fluorescent Z′ measures reflects the inherent biology of apoptosis, not assay performance in HTS environments.

**Table 1:** High throughput assay quality as a function of time with bortezomib-induced K562

Exposure (H)	PS exposure		Secondary necrosis	
	Induction ratio	Z′	Induction ratio	Z′
10	2.3	0.14	> 2	> 0.01
11	2.8	0.34	> 2	> 0.01
12	3.5	0.44	> 2	> 0.01
13	4.4	0.54	> 2	> 0.01
14	5.4	0.59	> 2	> 0.01
15	6.2	0.61	> 2	> 0.01
16	7.1	0.64	> 2	> 0.01
17	8.0	0.66	2.2	0.01
18	8.8	0.69	2.4	0.15
19	9.6	0.7	2.7	0.26
20	10.0	0.71	3.0	0.37
21	10.4	0.72	3.4	0.43
22	10.9	0.73	3.8	0.49
23	11.4	0.75	4.2	0.55
24	11.4	0.75	4.8	0.56
48	2.8	0.41	16.9	0.84

### Annexin V method comparison: homogenous microtiter plate versus flow cytometry

**Fig. 6:**
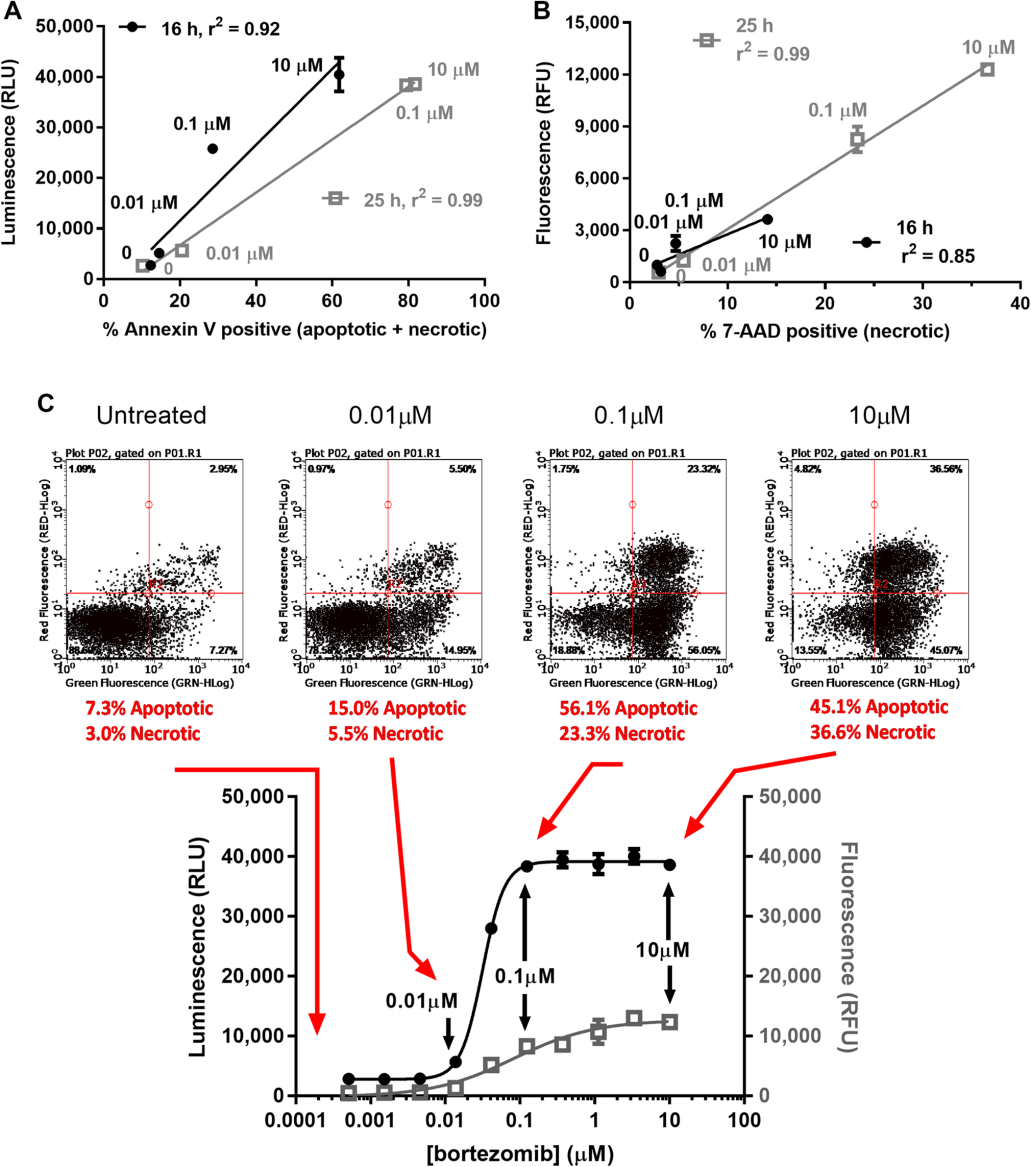
Correlation between the plate-based, multimode reader method and conventional flow cytometry. Luminescence (**a**) produced by PS exposure and fluorescence (**b**) associated with secondary necrosis were plotted versus their respective orthogonal flow cytometry measures after 16 and 25 h of bortezomib exposure on K562. Scatter plot data collected at four treatment conditions is superimposed on the full dose–response relationship collected using the bioluminescent, real-time format at 25 h (**c**). 10,000 events were collected for each histogram. n = 1 for flow cytometry sample analysis and n = 4 for plate method

A majority of annexin V assay users utilize flow cytometry to measure PS-positive and necrotic populations. We sought to directly benchmark the data from the plate-based assay to data collected using a fluorescently labelled annexin V probe and a spectrally compatible, dead cell dye analyzed by flow cytometry. After 16 h of bortezomib exposure, K562 cells demonstrated a dose-dependent increase in PS-dependent luminescence with only modest increases in secondary necrosis-related fluorescence (data not shown). This correlated reasonably well with the flow cytometry data (PS exposure: r^2^ = 0.92 and membrane integrity: r^2^ = 0.85) (Fig. 6a, b) considering technical differences resident in sample preparation and analysis for the respective methods. An improved correlation for both biomarkers emerged at 25 h (PS exposure: r^2^ = 0.99 and membrane integrity: r^2^ = 0.99) as the apoptotic phenotype matured in the treated populations. At this time point, a nominal dosage of 0.01 µM produced twofold increases in the annexin V-stained flow population relative to untreated control with a commensurate twofold increase in luminescence in the plate-based format (Fig. 6c). With additional tenfold increases in bortezomib, the annexin V flow cytometry and plate data demonstrated annexin V-positive plateaus in their respective readouts, again consistent with a fully potentiated and mature apoptotic response. Last, the secondary necrotic population was faithfully represented in both assay formats.

### Comparison to caspase-3/7 activation

**Fig. 7:**
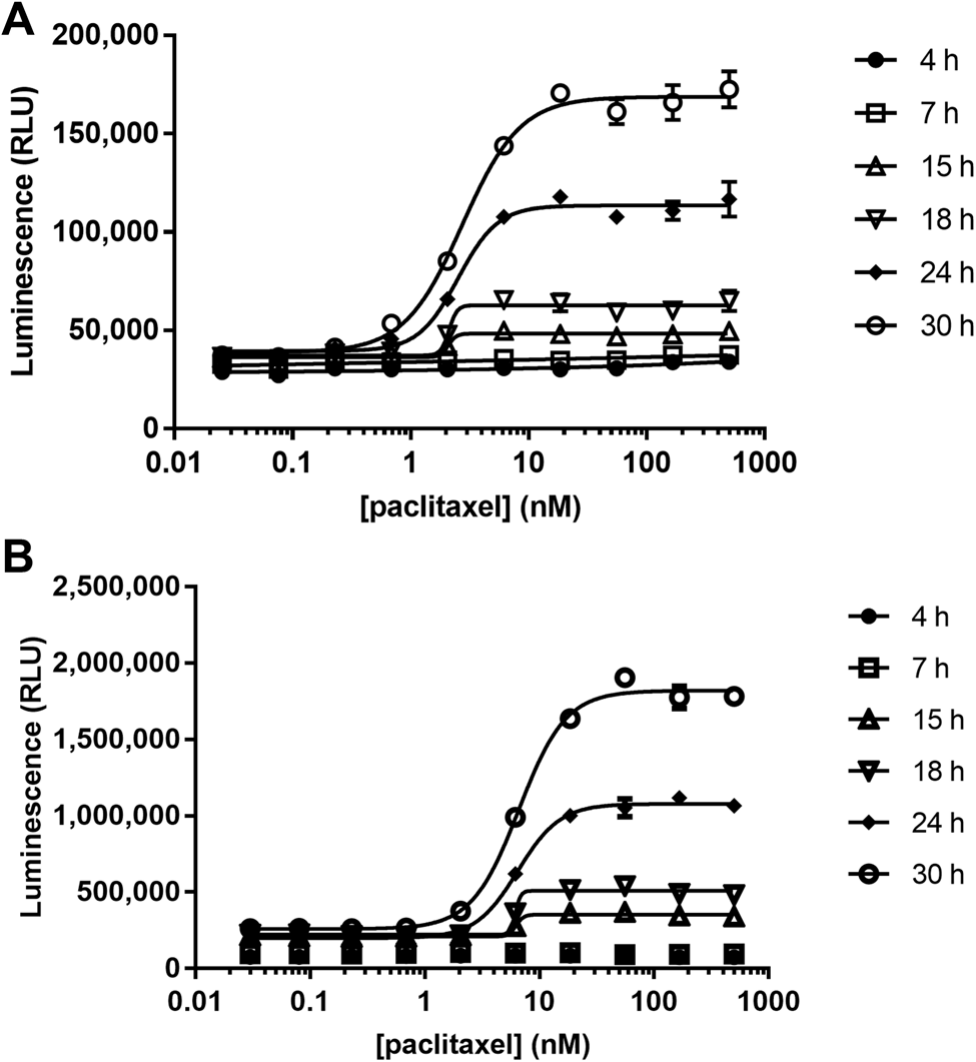
A kinetic comparison between PS exposure and caspase activation after paclitaxel treatment of HepG2. Annexin-binding luminescence was collected by sequential measurements of the same plate over the 30 h time course (**a**), whereas caspase activity data was collected from parallel plates using lytic endpoints (**b**). n = 4 for each dose

Caspase activation is an early and definitive biomarker for apoptosis but is typically detected using lytic endpoint formats. We conducted parallel experimentation to formally examine the kinetics of caspase activation versus PS exposure using HepG2 cells stimulated with paclitaxel (Fig. 7). At the time points examined, PS exposure and caspase activity appeared to be relatively concordant in terms of magnitude of response and potency. The caspase activity assay produced a notable tenfold difference in luminescent intensity but had a proportionally elevated untreated background and, therefore, enjoyed no tangible benefit in induction signal-window ratios.

### Bioluminescent imaging

**Fig. 8:**
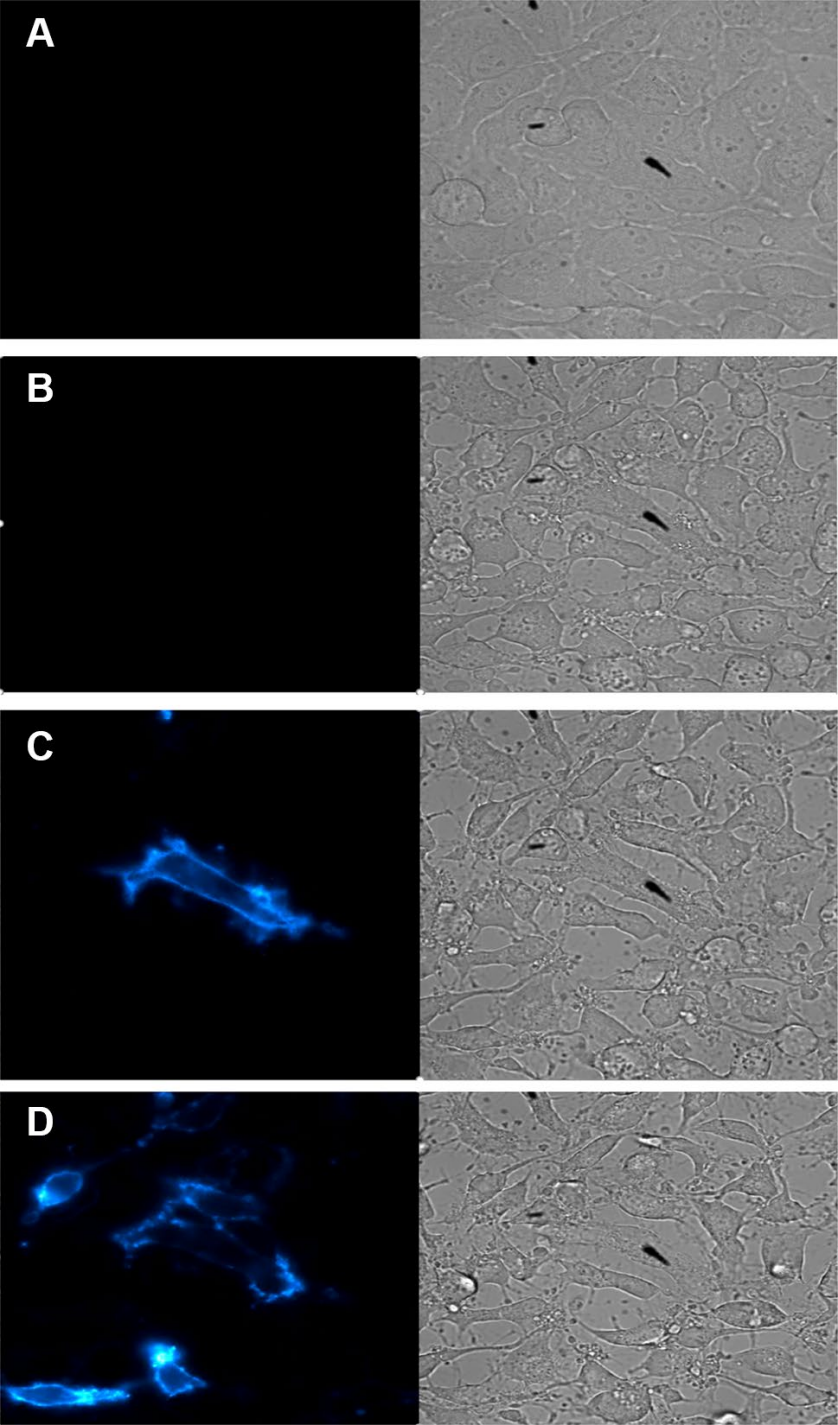
Bioluminescent imaging of the apoptotic response. An Olympus LV200 was used to collect phase-contrast and luminescence associated with PS exposure over a 6-h time course of HeLa treated with 2 µM staurosporine. Time zero (**a**), 3 h (**b**), 3.7 h (**c**) and 6 h (**d**) images shown

High-content imaging platforms utilizing fluorescent annexin conjugates allow for morphological co-localization of the probe with PS at the cell surface. Unfortunately, these images are typically collected at static endpoints. We sought to verify proper localization of our annexin NanoBiT™ fusions while investigating the relative density and kinetics of PS exposure using real-time bioluminescent imaging. In our model, attachment-dependent HeLa cells treated with staurosporine demonstrated noteworthy retraction from the plate surface and other ill-defined morphological changes at 3 h in the brightfield channel but produced no luminescence associated with PS exposure. By 3.7 h, however, a halo of luminescence (pseudo-colored blue) surrounded an elongated cell undergoing apoptosis (Fig. 8). With continued staurosporine exposure (6 h), more cells developed luminescent staining associated with nascent PS-containing lipid rafts in their outer membranes. Although the degree of PS staining was largely static, the bioluminescent images also appeared to reveal a certain degree of lateral fluidity which was consistent with other reports of dynamically remodeled membranes during the death process [24].

## Discussion

The in vitro cell death response is dictated by both the potency of the cytotoxic stimulus and the length of the exposure period. Common and conventional endpoint chemistries are often employed after an exposure to determine the potency and magnitude of the effect. Unfortunately, many cytotoxicity markers are unstable in the extracellular milieu and, therefore, can lead to a substantial underestimation of the actual cell death response in extended incubations. Endpoint viability assays are less encumbered by the experimental constraints of exposure because they measure the level of retained biomarkers, but also reveal substantially less information about the response other than the number of remaining viable cells.

It has become increasingly clear that any characterization of in vitro cytotoxic potential or liability must include kinetic measurements to get a better understanding of the onset, duration and progress of cytotoxic effects. As such, a number of real-time viability and cytotoxicity assay formats have been developed and utilized. In the simplest and most accessible formats, optimized pro-fluorescent or -luminescent probes are delivered directly to cells at the time of seeding or dosing and remain in the cultures throughout the drug exposure or treatment period [25, 26]. When relative fluorescence and/or luminescence data are collected at scheduled time points or continuously measured (via atmospheric control of multimode reader) over the exposure, inflections in signals can reveal the initiation, progression, and completion of cytotoxicity responses. In broad dosing scenarios involving logs of compound dilutions, standard cytotoxic EC_50_ can be obtained at any time point during the exposure, but additional insight can be gathered regarding doses producing acute cytostasis.

Cytotoxic potency and potential alone are important considerations for any compound, treatment or agent, but ultimately, defining the manner by which an agent or compound initiates a particular cell death program portends its pharmacological future. It is now well-noted that several cell death fates and pathways exist. Unless specific motivation exists for identifying and defining alternative death mechanisms, apoptosis is considered to be the most desirable cell death phenotype to elicit due to it being typically more immunologically and therapeutically tolerable [27].

Multiple endpoint options exist for characterizing apoptotic responses, but to date, only a few methods have been described which gather real-time features of the program’s progression [28, 29]. We sought a well-validated and tractable marker and related non-destructive methods for measuring the apoptotic phenotype and ultimately focused our efforts on means relating to measuring PS exposure. With direction from Dixon et al., and their work related to the creation of a newly engineered binary luciferase, we undertook efforts to develop a homogeneous, real-time apoptosis assay that produces data distinct from other mechanistic forms of cell death.

Creation of useful recombinant Annexin V-NanoBiT™ fusion proteins necessary for the real-time apoptosis assay required a combination of informed design and trial and error. Using standard molecular biology techniques, DNA constructs were engineered to express genetically encoded proteins containing full-length annexin V, an affinity purification tag sequence, and either the LgBiT™ or SmBiT™ luciferase subunits [19]. In order to increase the likelihood of optimal spatial-orientation during the PS-binding event and structural complementation, four distinct proteins were created which contained the NanoBiT™ fusion subunits at either the amino- or carboxyl-terminal positions. Ultimately, the amino-terminal fusions produced the optimal assay performance profile. Although several peptides have been developed to facilitate NanoBiT™ complementation, the lower affinity SmBiT™ peptide was chosen because of its inherent structural integrity in cell-based environments, as well as its lack of influence on proximity complementation initiated and potentiated by its fusion partner. In our real-time annexin V apoptosis assay, the modest affinity of SmBiT™ for LgBiT™ assures low spontaneous complementation in the absence of PS exposure and allows for full, functional reversibility.

Once purified to greater than 95% purity, the recombinant fusion proteins were tested and confirmed to contain essentially undetectable levels of endotoxin contaminants. This purity allowed for robust and reproducible quantitation and greatly mitigated the physiological impact of the reagent grade proteins in real-time, cell culturing formats.

The relative topology and density of PS exposed on the apoptotic membrane interface has been studied extensively with native and derivitized annexin V-related proteins [30, 31]. Whereas native annexin V has been shown to trimerize into highly ordered oligomers on PS rafts, it was unknown how introduction of binary luciferase subunits to the annexin V protein would influence optimal PS binding and complementation stoichiometry [32]. A simple matrix titration of the fusion proteins revealed an expected concentration dependence for optimal pairing performance as well as a semi-rigid requirement for near-equivalence in stoichiometry. It is notable that the concentration of annexin V fusion pairs necessary for optimal performance in this bioluminescent format is substantially (an order or more of magnitude) less than is required for optimal fluorescent annexin V staining technologies. This reduced requirement for annexin V protein content, as a reagent, is likely possible due to the high sensitivity of the reconstituted NanoBiT™ enzyme yet appears to be at a saturating level for measuring the proportionality of PS exposure in all the cells models tested (unpublished data). This information assuaged our concerns that creation of NanoBiT–annexin V fusions may adversely impact binding affinity.

Luciferase reporter assay systems require an enzyme substrate to generate photons of light. In endpoint formats, the reporting molecule is detected by the addition of a reagent which has been configured to deliver a substantial molar excess of substrate in an optimized buffer system to produce “glow type” luminescence [33]. The enzymatic constraints for a real-time, cell health assay format are more appreciable. In this case, the substrate must be available and non-limiting during the entire exposure time course, compatible with a variety of culture media, and exhibit physiologic tolerability.

Our previous cell-based assay experiences with a novel esterase-cleavable, pro-furimazine compound (endurazine) suggested that it may enable a homogeneous and prolonged real-time, bioluminescent annexin V assay format [34, 35]. Cognizant of the potential for variation in inherent esterase activity levels found in the variety of cell types that might be studied using the assay, we sought to better understand the cell-dependent substrate de-protection step over the course of a typical compound exposure. Our studies revealed that esterase-dependent de-protection begins immediately upon addition of the reagent and typically reaches steady state between release and decay within 2–4 h at 37 °C. Therefore, the bioluminescent annexin reagent components benefit from pre-incubation with cells prior to induction of apoptosis with fast-acting inducers for optimal detection fidelity.

Once steady state is achieved, the substrate de-protection/consumption dynamic provides a stable and saturating pool of furimazine, and thus light proportional to the degree of PS exposure during a normal 48 h assay time course. When cells die by apoptosis or other cell death programs, the esterase released by loss of membrane integrity provides an additional time period of sustained de-protection. This allows for a period of glow luminescence even when the cell death program is fully potentiated. This prolonged signal maintenance affords enhanced robustness and operator flexibility with regard to measuring the kinetics of PS exposure via apoptosis. In cases of profound cytotoxicity, where no viable bystander cells afford pro-substrate de-protection, however, luminescent signal will decay as a function of released esterase enzyme lability.

To test the utility of the assay reagent under standard operational conditions, we dosed cells with an agent which produces rapid apoptosis (staurosporine) and a compound that initiates apoptosis at a later time after provoking DNA damage (paclitaxel). In both instances, clear exposure and dose dependence were observed for PS appearance (luminescence) and membrane integrity loss (fluorescence). Notably, apoptosis inducers produce substantial luminescence signal increases (above untreated background) prior to gains in fluorescence. This characteristic temporal lag is consistent with the phenotypic surface exposure of PS prior to loss of membrane integrity during apoptosis. Interestingly, other forms of mechanistic cell death, such as primary necrosis and necroptosis, demonstrate concurrent increases in luminescence and fluorescence. Although all mechanism of action determinations should be confirmed with orthogonal methodologies, these luminescence and fluorescence signal “signatures” can be used to tentatively identify mode of cell death (unpublished observations).

After establishing general tolerability of the real-time reagent with untreated healthy cells, we strived to determine whether the real-time reagent exerted any improper biological influences on treated cells. Our collective experiences with at least 20 cell lines from divergent lineages, with numerous inducing agents, suggest the reagent is tolerable without significant detriment (unpublished observation). When influences were observed, they were minor and somewhat contradictory with respect to influencing the magnitude and progression of apoptosis. Van Genderen et al. suggest that any observable effects from annexin assay components are cell-type and cytotoxin-dependent [36]. Therefore, real-time annexin assay users should be vigilant for the possibility of unique cell lineage sensitivities. Often, detrimental effects can be eliminated or greatly reduced by decreasing the concentration of CaCl_2_ and/or the Annexin V NanoBiT™ substrate; however, these reagent reductions will lower the overall luminescence intensity and signal sustainability over time (unpublished observation).

The ultimate utility of any plate-based assay depends upon scalability into high-density microplate formats. Scalability requires a reagent formulation that can be accurately dispensed using robotic liquid handlers and can support robust, invariant signal windows necessary for identifying screening “hits”. During development and validation of the complete luminescent assay system, intra- and inter-assay variability were routinely between 5 and 10%. The real-time annexin V assay provided these basic attributes but is also uniquely capable of addressing the inherent unpredictability of apoptosis induction kinetics with unknown new chemical entities. In other words, the real-time (or scheduled) collection of data allows for meaningful evaluation of the apoptotic induction potential of each unknown compound within the context of its own kinetic behavior. Previously, such data would have only been possible with multiple parallel processed plates over a time course. The benefits of improved kinetic resolution and the economy of avoiding unnecessary duplicative effort are therefore evident using the real-time methodology.

Scientific due diligence often requires apparent apoptotic responses to be independently corroborated using orthogonal methods. Our efforts demonstrate that the plate-based, real-time annexin V assay delivers results that are fundamentally similar to flow cytometry analyses using fluorophore-labeled annexin V. Minor discordance between the methods early in the apoptotic response are likely attributable to technical differences in sample preparation. For instance, fluorescent annexin V methods require washing and removal of unbound probe obviated by the luminescent method. Therefore, it is possible that PS-containing extracellular vesicles are detected by the luminescent method but eliminated from flow cytometry analysis. With the real-time method, the magnitude of responses (in either luminescence or fluorescence channels) serve as surrogates for the percentage of cells that have exposed PS or have lost membrane integrity. A significant advantage of the real-time assay is that compound effects can be monitored over time using a single sample (per concentration), in contrast with conventional assays which require a separate sample for each time point. Further considerations relating to the inequities in instrument costs, maintenance, operator availability and accessibility can be argued in favor of use of the more accessible real-time method.

Caspase activity detection methods have historically proven extremely useful for defining apoptotic response profiles. Our formal comparison with a highly-sensitive and well-validated bioluminescent, caspases 3/7 assay method demonstrates general concordance with the real-time annexin assay method in terms of potency and magnitude of response. It is now appreciated that caspase activation is an early and necessary event for apoptotic PS exposure. Therefore, early in apoptotic responses, caspase activity may be measurable in the absence of notable PS exposure signals. Caspase activity is notoriously transient, however, and subject to substantial enzymatic degradation as the cell death program progresses. Therefore, it is often necessary to deploy caspase activity assays in time course formats using multiple assay plates. Interestingly, real-time annexin assay data can be collected as described previously, then followed after the onset of PS exposure by an endpoint caspase activation assay allowing for same-well orthogonal measures of apoptosis. This multiplexed method is possible because lytic agents in the caspase reagent disrupt PS–annexin binding and effectively quench luminescence from the annexin V assay.

Imaging methods for apoptosis have traditionally focused on fluorescent annexin cell-labeling techniques due to the general availability of robust reagents, probes and well-established protocols. New imaging approaches utilizing polarity sensitive probes conjugated to IANBD dyes and caspase-3 sensitive substrates have enabled real-time functionality [11, 12, 37]. With the expanding availability of new bioluminescent imaging systems, real-time bioluminescent apoptosis imaging possibilities now exist. We chose to explore how the luminescent annexin V reagent would perform in an imaging modality both from a practical and technical perspective. Imaging proved possible in real-time, provided pH control was maintained using a CO_2_-independent medium in the absence of exogenously perfused gases. The imaging provided striking and tangible links between changes in morphology and the transition to PS exposure in a number of model systems. Obvious applications utilizing mixed-cell lineages and immune effector and target incubations are being developed in our lab and will continue to be explored.

In conclusion, the real-time, bioluminescent annexin V assay method described here provides the full utility and functionality of conventional annexin V analysis methods but requires substantially less operator effort and can be conducted using standard plate-based measures. The time-resolved nature of the assay allows for enhanced scrutiny of the relationship between dose-dependent cytotoxic effects and exposure and reveals putative mechanism of action.

## References

[1] Elmore S (2007). Apoptosis: a review of programmed cell death. Toxicol Pathol.

[2] Norbury CJ, Hickson ID (2001). Cellular responses to DNA damage. Annu Rev Pharmacol Toxicol.

[3] Fadeel B, Orrenius S (2005). Apoptosis: a basic biological phenomenon with wide-ranging implications in human disease. J Intern Med.

[4] McGahon A, Martin S, Bissonnette R, Mahboubi A, Shi Y, Mogil R, Green D (1995). The end of the (cell) line: methods for the study of apoptosis in vitro. Methods Cell Biol.

[5] Majno G, Joris I (1995). Apoptosis, oncosis, and necrosis. An overview of cell death. Am J Pathol.

[6] Levin S, Bucci T, Cohen S, Fix A, Hardisty J, LeGrand E, Maronpot R, Trump B (1999). The nomenclature of cell death: recommendations of an ad hoc Committee of the Society of Toxicologic Pathologists. Toxicol Pathol.

[7] Riss T, Moravec R (2004). Use of multiple assay endpoints to investigate the effects of incubation time, dose of toxin, and plating density in cell-based assays. Assay Drug Dev Technol.

[8] Niles A, Moravec R, Riss T (2009). In vitro viability and cytotoxicity testing and same-well multiparametric combinations for high throughput screening. Curr Chem Genom.

[9] Niles A, Moravec R, Riss T (2008). Update on in vitro cytotoxicity assays for drug development. Exp Opin Drug Discov.

[10] Sener L, Albeniz G, Dinc B, Albeniz I (2017). iCELLigence real-time cell analysis system for examining the cytotoxcity of drugs to cancer cell lines. Exp Ther Med.

[11] Kim Y, Chen J, Chan J, Langen R (2010). Engineering a polarity-sensitive biosensor for time-lapse imaging of apoptotic processes and degeneration. Nat Methods.

[12] Kim Y, Chen J, Langen R, Chan J (2010). Monitoring apoptosis and neuronal degeneration by real-time detection of phosphatidylserine externalization using a polarity-sensitive indicator of viability and apoptosis. Nat Protoc.

[13] Martin SJ, Reutelingsperger C, McGahon A, Rader J, Van Schie RC, LaFace D, Green D (1995). Early redistribution of plasma membrane phosphatidylserine is a general feature of apoptosis regardless of the initiating stimulus: inhibition by overexpression of Bcl-2 and Abl. J Exp Med.

[14] Birge RB, Boeltz S, Kumar S, Carlson J, Wanderley J, Calianese D, Barcinski M, Brekken R, Huang X, Hutchins JT, Freimark B, Empig C, Mercer J, Schroit A, Schett G, Herrmann M (2016). Phosphatidylserine is a global immunosuppressive signal in efferocytosis, infectious disease, and cancer. Cell Death Differ.

[15] van Meer G, Voelker DR, Feigenson GW (2008). Membrane lipids: where they are and how they behave. Nat Rev Mol Cell Biol.

[16] van der Mark VA, Elferink R, Paulusma CC (2013). P4 ATPases: flippases in health and disease. Int J Mol Sci.

[17] Koopman G, Reutelinsperger C, Kuijten G, Keehnen R, Pals S, van Oers MH (1994). Annexin V for flow cytometric detection of phosphatidyl serine expression on B cells undergoing apoptosis. Blood.

[18] Concha NO, Head JF, Kaetzel MA, Dedman JR, Seaton BA (1992). Annexin V forms calcium dependent trimeric units on phospholipid vesicles. FEBS Lett.

[19] Dixon AS, Schwinn MK, Hall MP, Zimmerman K, Otto P, Lubben TH, Butler BL, Binkowski BF, Machleidt T, Kirkland TA, Wood MG, Eggers CT, Encell LP, Wood KV (2016). NanoLuc complementation reporter optimized for accurate measurement of protein interactions in cells. ACS Chem Biol.

[20] Niles A, Riss T (2014). Multiplexed viability, cytotoxicity, and caspase activity assays. Methods Mol Biol.

[21] Zhang JH, Chung TD, Oldenburg KR (1999). A simple statistical parameter for use in evaluation and validation of high throughput screening assays. J Biomol Screen.

[22] Gidon-Jeangirard C, Hugel B, Holl V, Toti F, Laplanche J, Meyer D, Freyssinet J (1999). Annexin V delays apoptosis while exerting an external constraint preventing the release of CD4 + and PrPc + membrane particles in a human T lymphocyte model. J Immunol.

[23] Monceau V, Belikova Y, Kratassiouk G, Charue D, Camors E, Communal C, Trouve P, Russo-Marie F, Charlemagne D (2004). Externalization of endogenous annexin A5 participates in apoptosis of rat cardiomyocytes. Cardiovasc Res.

[24] Jessel R, Haertel S, Socaciu C, Tykhonova S, Diehl HA (2002). Kinetics of apoptotic markers in exogenously induced apoptosis of EL4 cells. J Cell Mol.

[25] Duellman S, Zhou W, Meisenheimer P, Vidugiris G, Cali JJ, Gautam P, Wennerberg K, Vidugiriene J (2015). Bioluminescent, non-lytic, real-time cell viability assay and use in inhibitor screening. Assay Drug Dev Technol.

[26] Chiaraviglio L, Kirby JE (2014). Evaluation of impermeant, DNA-binding dye fluorescence as a real-time readout of eukaryotic cell toxicity in a high throughput screening format. Assay Drug Dev Technol.

[27] Fink S, Cookson B (2005). Apoptosis, pyroptosis, and necrosis: mechanistic description of dead and dying eukaryotic cells. Infect Immun.

[28] Gasser JP, Hehl M, Millward TA (2009). A homogeneous time-resolved fluorescence resonance energy transfer assay for phosphatidylserine exposure on apoptotic cells. Anal Biochem.

[29] Meyer-Almes FJ (2005) Chemosensitivity determination using phosphatidylserine. US Patent 6,9499,350

[30] Tait JF, Gibson D (1992). Phospholipid binding of annexin V: effects of calcium and membrane phosphatidylserine content. Arch Biochem Biophys.

[31] Tait JF, Smith C, Wood BL (1999). Measurement of phosphatidylserine exposure in leukocytes and platelets by whole-blood flow cytometry with annexin V. Blood Cells Mol Dis.

[32] Oling F, Bergsma-Schutter W, Brisson A (2001). Trimers, dimers of trimers, and trimers of trimers are common building blocks of annexin A5 two-dimensional crystals. J Struct Biol.

[33] Fan F, Wood KV (2007). Bioluminescent assays for high-throughput screening. Assay Drug Dev Technol.

[34] Klaubert DH, Meisenheimer P, Unch J (2014) Imidazo[1,2-α]pyrazine derivatives. US Patent 8,809,529

[35] Schwinn M, Machleidt T, Zimmerman K, Eggers C, Dixon A, Hurst R, Hall M, Encell L, Binkowski B, Wood K (2017). CRISPR-mediated tagging of endogenous proteins with a luminescent peptide. ACS Chem Biol.

[36] van Genderen HO, Kenis H, Hofstra L, Narula J, Reutelingsperger C (2008). Extracellular annexin A5: functions of phosphatidylserine-binding and two-dimensional crystallization. Biochem Biophys Acta.

[37] Cen H, Mao F, Aronchik I, Fuentes R, Firestone G (2008). DEVD-Nucview488: a novel class of enzyme substrates for real-time detection of caspase-3 activity in cells. FASEB J.

